# Supplementary material for: Study protocol for a randomized clinical pilot trial investigating feasibility and efficacy of augmenting a virtual reality-assisted intervention targeting auditory verbal hallucinations with biofeedback: The Neuro-VR study
Source: PLoS One. 2026 Feb 26;21(2):e0333716. doi: 10.1371/journal.pone.0333716 (PMC12944719; doi:10.1371/journal.pone.0333716)
Supplement: S2 File — The latest version of the protocol approved by the National Committee on Health Research Ethics for the Capital Region of Denmark (Version 5.0, 14. April 2025, H-24010871). (PDF) [file pone.0333716.s002.pdf]

The Neuro-VR study: a pilot study investigating an innovative virtual reality-based intervention employing biofeedback to increase tolerability and therapy efficacy in psychosis

Neuro-VR: et pilotstudie til undersøgelse af en innovativ virtual-reality baseret intervention med biofeedback til at øge effekt og tolerabilitet ved psykose

Forsøgsprotokol

Version 5.0.

Forsøgsleder Louise Birkedal Glenthøj, dr.med, PhD, specialpsykolog, specialist i psykoterapi, lektor og forskningsleder VIRTU Research Group, Psykiatrisk Center København, Københavns Universitet

Indhold

Aim 4
Background and rationale 4
2.2.Biofeedback 5
2.3 Hypotheses 6
Methods 7
Interventions 7
Adverse events 8
Outcomes 8
Randomization 9
Statistical analysis 9
Ethical Considerations 10
Dissemination 10
Feasibility 10
Potential impact 11
Timeline 11
Dansk tillæg til forsøgsprotokol 12
Informeret samtykke og rekruttering 12
Kontakthyppighed 13
Kriterier for diskontinuation 14
Procedure for discontinuation 14
Undersøgelser i studiet 14
Symptom- og funktionsniveau 14
EEG-undersøgelse 14
Tidsforbrug 15
Behandlingsmodaliteter 15
Medicinering 15
Forsøgets bivirkninger, risici og ulemper 15
Databehandling 16
Journaloplysninger 16
Erstatningsordning 18
Videnskabsetisk redegørelse 18
Fordelene for patienterne 18
Ulemperne for patienter 18

Studiets potentiale 19
Økonomiske forhold og klausuler 19
Initiativtager 19
Vederlag 20
Offentliggørelse 20
Organisation 20
Gennemførlighed 20

Aim

We aim to pioneer the utilization of Electroencephalography (EEG), Galvanic Skin Response (GSR), Photoplethysmography (PPG) and Electrocardiography (ECG) based Biofeedback (BF) within the innovative virtual reality-based framework for treating auditory verbal hallucinations (AVH) in psychosis. A distinguishing feature of this approach is the real-time acquisition of BF on patient brain activity during therapy sessions and deviates from conventional psychiatric paradigms that seldom rely on immediate physiological metrics in guiding therapy. By incorporating BF into the therapeutic procedure, we envisage enhancing the precision, personalization and efficacy of the treatment based on individual physiological responses. Furthermore, the investigative focus on the neural underpinnings responsible for AVH attenuation will pave the way for discerning patient subgroups with varied therapy responsiveness.

Background and rationale

Schizophrenia Spectrum Disorders (SSD) are among the most severe and incapacitating mental disorders affecting over 24 million people globally (1). The illness often develops at a young age, and tends to persist throughout the individual's lifetime, causing substantial distress to the sufferers, their families, and the broader society (2). AVH are the most salient and debilitating symptom in SSD and related conditions (3). Pharmacotherapies are effective only in approximately 70% of patients, yet, posing mild to moderate side effects (4,5), which constitute one of the major determinants of nonadherence potentially leading to a psychotic relapse (4). The economic repercussions of schizophrenia are equally substantial with the financial burden in Europe equal to €93.9 billion (6).

Cognitive Behavioral Therapy for psychosis (CBTp) (7) is the gold standard intervention for psychosis, that has, however, proven mild to moderate effect sizes (8). This has spurred interest into the development of targeted and shorter therapies for AVH (9). Several novel approaches focus on AVH as identity-bearing entities with whom patients establish submissive relationships that may reflect broader social dynamics (10). In line with this, the so-called avatar therapy (AT) has emerged as a part of this new generation of relational treatments that integrates phenomenological experiences of individuals perceiving voices into the therapeutic framework. Early studies, such as those by Leff et al. (11) and the AT Trial (9), have demonstrated AT's potential in mitigating AVH severity. More recent experiments involving immersive VR psychotherapy have noted even more substantial improvements in AVH, depression symptoms, and overall quality of life (12,13).

Our research group, VIRTU, has pioneered a fully immersive VR-based intervention, with preliminary results indicating benefits. Two major ongoing studies, including ours, the CHALLENGE trial (14,15), are further probing the efficacy of VR therapy for AVH. Initial findings from these studies suggest remarkable patient improvement, emphasizing the imperative to deeply understand the therapy's impacts (15). Merging BF with

VRAT offers groundbreaking insights into the dynamics of therapeutic processes, and promotes the tailoring of treatment based on neuroscientific informed mechanisms of change. This combination not only deepens our knowledge of VRAT's neural implications on AVH but also refines treatment strategies

Incorporating BF into VRAT offers transformative insights into therapy mechanisms, while facilitating treatment personalization. This real-time neuro-monitoring technique informs therapy intensity, enhancing individualized approaches and bolstering therapeutic alliance. Such integration will deepen our comprehension of VRAT's neural intricacies and its effects on AVH. Our research will aspire to refine interventions for AVH sufferers, advancing evidence-based healthcare quality.

2.2. Biofeedback

Biofeedback is a technique that uses biosensors and electronic devices to monitor and display physiological responses in real time (16,17). By using sensors, such as electrodes, individuals can gain insight into how their body reacts to stress or anxiety. The goal is to enhance conscious self-regulation of physiological processes that typically occur outside of awareness. In our study, we will include the following non-invasive methods: Electroencephalography (EEG) in a neurofeedback (NF) setup, Galvanic Skin Response (GSR), Photoplethysmography (PPG) and Electrocardiography (ECG) .

EEG-based NF has demonstrated a significant potential in addressing multiple psychiatric disorders, including anxiety (15), depression (18), attention deficit hyperactivity disorder (ADHD) (19), and notably schizophrenia (20). NF offers real-time feedback on brain activity related to affective or behavioral states underscoring the method's therapeutic aspirations (21), and has been shown to induce structural brain changes (https://pubmed.ncbi.nlm.nih.gov/23536382/). Compared to Functional Magnetic Resonance Imagery (fMRI), EEG provides a more cost-effective alternative in shaping NF protocols (22).

Specifically, for schizophrenia and AVH, EEG provides high temporal resolution, capturing rapid shifts in brain activity (23,24). Different brain waves frequencies such as delta (δ: 0.5–4 Hz), theta (θ: 4–8 Hz), alpha (α: 8–13 Hz), beta (β: 13–30 Hz), and gamma (γ: 30–150 Hz) waves (25), play essential roles in diverse cognitive functions. Functions related to AVH in schizophrenia can be closely monitored using EEG. While there are challenges like limited spatial resolution (25) and the presence of unwanted non-brain signals or 'noise' potentially confounding the interpretation of data (26,27) , innovative solutions are being developed that address these limitations aiming to make EEG more user-friendly and efficient (28).

Recent research has highlighted the importance of EEG in uncovering neural roots of AVH and schizophrenia symptoms (29) Specifically, variations in heightened alpha, beta (29,30), delta, and theta

oscillations (31) have been associated with intense emotional manifestations of AVH (32). Therefore, these indications may guide the therapist in directly addressing and potentially modulate brain activity during the sessions. Initial case studies have elucidated first evidence of potential benefits of EEG-based NF in schizophrenia (33). Following, a recent feasibility study has confirmed preliminary evidence of EEG-based NF as a viable therapeutic option, specifically for AVH in schizophrenia (20). However, further research is needed to solidify the effects.

GSR is a non-invasive method that has been widely used as a surrogate marker of the autonomous nervous system activity in various psychophysiological applications, including emotional arousal (34–37), stress (38–41) and panic disorder (42). It reflects changes in the skin's electrical conductivity in response to eccrine sweat gland activity, which is typically measured with two closely placed electrodes on the fingers of the participant. Skin conductivity is strongly associated with arousal levels and is a fundamental metric when intending to predict anxiety (34,43). The literature further indicates that increases in heart rate are also highly correlated with anxiety (44) and provide a reliable measure for real-time assessments (43). Heart rate can be measured using different technologies, including ECG and PPG. ECG is a well-established method that records the heart's electrical activity via electrodes placed on the skin, offering high accuracy in heart rate monitoring (45). PPG, on the other hand, is an optical technique that estimates heart rate based on blood volume changes in the microvascular tissue, typically measured on the fingers or earlobe (43). The combination of VR and biofeedback has been proven to be feasible in previous studies (46). The biofeedback measures are completely non-invasive, utilizing electrodes placed on the participant's fingers and a heart rate monitor belt positioned around the chest.

While the intersection of VR and BF offers exciting possibilities for mental health interventions, this is a relatively new area of research, with only a few completed studies treating conditions such as anxiety disorders (47–50), ADHD (51), and Autism Spectrum Disorder (ASD) (52), its application in schizophrenia, especially in relation to AVH, remains unexplored. A recent endeavor combined VR and EEG approach to assess sensory information processing in patients with schizophrenia (53). The successful implementation of this combined approach provides a foundation for further investigations into integrating VR and EEG technologies for patients with SSD.

2.3 Hypotheses

We hypothesize that, in patients with schizophrenia:

1) VRAT supplemented with BF will be feasible and acceptable

2) VRAT supplemented with BF will provide indications for superiority compared to VRAT in reducing AVH, improving daily-life functioning, and quality of life.

Methods

This pilot study will enroll 30 patients with schizophrenia from the psychiatric outpatient clinics in the Capital Region of Denmark. Participants will be randomized to either 8 sessions of a standard VRAT or VRAT supplemented with BF. Patients will be assessed at baseline and treatment cessation (3-months).

Participant Selection: Patients aged 18-65 with a diagnosis of SSD based on ICD-10 (codes: F20, 22-23, 25-29), and clinically relevant AVH within at least the past 3 months, corresponding to SAPS score of 3 or more and no changes in antipsychotic medication in the preceding four weeks and no planned changes in antipsychotic medication in the 12-weeks following inclusion in the project, will be recruited to take part in the study. Participants will be excluded if they have the diagnosis of organic brain disease, intellectual disability (IQ<70), a current diagnosis of substance dependance hindering engaging in the therapy, a command of Danish or English insufficient to undergo therapy, hear voices in a language the therapist does not speak, or unable to identify a dominant voice to work with.

Interventions

Both interventions will be in an 8-session manualized format and conducted by therapists experienced in delivering the VRAT intervention to the target group. Both interventions follow the principles and manual of VRAT (described below), but the experimental intervention will add real-time BF that is expected to increase therapy tolerability and effectiveness.

VRAT Intervention: Immersive VRAT engages the patient in a direct dialogue with their auditory hallucination to change the power dynamics between the patient and the voices, and consequently empower the patient to increase control, power, and resilience towards the auditory hallucination (voice). In the initial session of treatment, with the use of a special VR Avatar program, the participants create a virtual avatar that corresponds to their visual perception that represents their hallucinated voice. Additionally, a voice transformation program transforms the voice of the therapist to match the hallucinated voice, as it is experienced by the patient. In the following sessions, the therapist initiates, encourages, and supports a virtual reality-based dialogue between the participant and the avatar (symbolizing the AVH).

Biofeedback: To process the physiological data and provide real-time feedback about the patient's mental state, we will implement a BF System integrated in the VR system, collecting data about the autonomic response during the therapy. BF is an operant conditioning paradigm that uses contingent rewards to motivate patients to produce specific patterns of brainwave or autonomic activity (17,51). The biofeedback will be shown on the therapist's screen and in the VR environment during the therapy sessions. Hence, our

system will give visual cues to both the therapist and patient about certain physiological changes connected to mental states. This way the therapist will be able to adjust the therapy according to the patient's individual needs (intensifying or lowering the difficulty of the exposure). Importantly, it allows for patients to identify and practice a downregulation of their autonomic activity related to distress in real time, consequently boosting the effect of the intervention.

As part of this study, we will develop an BF protocol that targets emotional regulation, auditory hallucinations, attention regulation, and normalizing brain activity in deviating regions. Through visual cues and guidance from the therapist, patients will learn techniques to regulate their brain activity. The targeted EEG signals will be displayed as a visual cue for therapists and patients represented by a thermometer system. To achieve the predefined, desired temperature range, the therapist will instruct the patient to self-regulate by moving the thermometer's indicator within that range.

Adverse events

Virtual reality therapy is generally well tolerated and with minimal or no side-effects or adverse events (54). There are, though, few reports of cyber sickness caused by virtual reality therapy. Side-effects and adverse events will be monitored and recorded throughout the study period.

Outcomes

Clinical outcome evaluations will take place at VIRTU Research Group by research assistants undergoing extensive training in the clinical assessments and receiving regular supervision.

Primary Outcomes: Primary outcomes include feasibility and acceptability to the therapy estimated as recruitment of ≥80% of the target sample in 12 months; ≥80% retention to study protocol at cessation of therapy (8 sessions) and ≥80% reporting a satisfaction rating of ≥7 on a Likert scale.

Secondary Outcomes: Scale for Assessment of Positive Symptoms (SAPS) will be used to assess eligibility of AVH. Different aspects of AVH will be measured with the Psychotic Symptoms Rating Scales. The Voice Power Differential Scale, The Voices Acceptance and Action, the Assertive Responding to Voices subscale and The revised Beliefs About Voices Questionnaire (BAVQ-R). Finally, the Personal and Social Performance Scale (PSP) and Social Functioning Scale will evaluate patients' social and daily functioning.

Exploratory Outcomes: Explorative outcomes are Brief Negative Symptoms Scale (BNSS), Self Evaluation of Negative Symptoms (SNS), Emotion Regulation Questionnaire (ERQ), the Difficulties in Emotion Regulation Scale (DERS), The Brief Core Schema Scales: Beliefs about self and others (BCSS), Client Satisfaction Questionnaire (CSQ), Simulator Sickness Questionnaire (SSQ), Calgary Depression Scale for

Schizophrenia (CDSS), Suicidal Ideation Attributes Scale (SIDAS), Childhood Trauma Questionnaire (CTQ),General Self-Efficacy, quality of life with WHO-5, Pittsburgh Sleep Quality Index (PSQI), Readiness for Therapy Questionnaire (RTQ), The Psychotherapy Motivation Questionnaire (Fragebogen zur Erfassung der Psychotherapiemotivation: FPTM) and the Birchwood Insight Scale (BIS)
A Natural Language Processing (NLP) approach will be used to elicit speech samples. NLP enables us to study disturbances related to thought, language and communication in psychosis. Finally, qualitative interviews with study participants will elucidate their experience with the intervention including the added BF component.

EEG Monitoring: EEG examinations will take place at Mental Health Center Glostrup by staff trained in EEG-procedures. Spontaneous EEG will be acquired at rest with the patient's eyes open and conducted at baseline and post-treatment. Based on previous research (20,55) we will particularly investigate generalised alpha band activity (8–12 Hz) changes as measured by quantitative EEG (qEEG), frontal alpha amplitude asymmetry between the left and right hemispheres (Fp1 and Fp2), changes in frontal/central/temporal delta (0.5–4 Hz) and theta (4–8 Hz) band power/coherence, qEEG in T3, T4 and T5, and beta coherence between the left and right temporal cortices (T3-T4). Additionally, the Prepulse inhibition of the startle reflex (PPI), P50 suppression, mismatch negativity (MMN).

Randomization

Randomisation will be centralized and computerized with a concealed randomisation. Block size will be unknown to the investigators and clinicians. The randomized intervention allocation is concealed until the statistical analyses of resulting data have been completed. Assessors engaged in outcome evaluation are blinded.

Statistical analysis

The study will use a mixed-methods design. Repeated measures analysis of variance (ANOVA) will be used to evaluate the differences in clinical symptoms and quality of life. We will assess trial procedures' feasibility by calculating proportions and 95% exact Clopper Pearson confidence intervals. This assessment covers feasibility and acceptability aspects such as recruitment, consent, participation, randomization and treatment retention. Furthermore, we will employ qualitative analyses to evaluate acceptability, considering participants' feedback on their therapy experience upon completing the treatment. The sample size of 30 participants is considered adequate for a pilot-study assessing feasibility, acceptability, and providing indications of intervention efficacy (56).

Ethical Considerations

Ethical approval will be obtained from the regional ethical committee
and the Danish Data Protection Agency before commencing the trial. We will comply with rules regarding data security and make sure that all the technical solutions comply with GDPR. Informed consent will be obtained from all participants, and their confidentiality and well-being will be protected throughout the study.

Dissemination

Study results will be disseminated at relevant conferences, in scientific journals to the public etc. Tentative papers derived from the study are: 1) Study protocol for NeuroVR: investigating feasibility and efficacy of augmenting a virtual reality-based intervention targeting auditory hallucinations with biofeedback. 2) Optimizing therapies for auditory hallucinations: results from the NeuroVR study investigating the effectiveness and tolerability of a virtual reality-based intervention utilizing real-time biofeedback. 3) Exploring neurophysiological correlates of response to a virtual reality-based intervention targeting auditory hallucinations: discerning patient subgroups with varied therapy responsiveness.

Feasibility

VIRTU Research Group, spearheaded by the applicant LBG, has extensive proficiency in conducting VR-based treatments in psychiatry through eight large-scale ongoing trials, comprising the psychosis population, and totaling to more than 1000 patients with psychiatric disorders. We have recently finalized enrollments of 266 patients with psychosis experiencing AVH and tested the VRAT intervention. The project group has a strong relationship with the clinical facilities in the capital region that readily refer patients to our research studies. It is therefore highly realistic to include the target number of 30 participants within the study period. Additionally, VIRTU has great expertise in completing all clinical and administrative documents needed for clinical trials along with proven capability of handling large datasets and performing planned analyses.
The software for VRAT will be delivered by Heka VR, the Danish company who developed the software specifically for use in treating AH in the Challenge trial (14). Heka will provide continuous technical support throughout study period.
The study is conducted in collaboration with Prof. Bjørn Ebdrup and senior researcher Tina Dam Kristensen, Center for Neuropsychiatric Schizophrenia Research, Mental Health Center Glostrup, who have extensive expertise in EEG-studies.

In conclusion, the proposed research project's feasibility is well-supported by the available resources, expertise, and patient population. The outlined timeline spans two years, encompassing distinct phases from preparation to knowledge dissemination.

Potential impact

The proposed study has the potential to revolutionize our understanding of mental health, particularly in how VR and BF impact AVH. By uncovering these mechanisms, we could tailor more effective therapeutic interventions, potentially improving treatments for conditions like schizophrenia. Combining BF and VR offers a potential shift in clinical protocols, allowing for interventions personalized to individual neurophysiology. Using real-time BF with VR can empower patients to self-regulate brain activity, possibly leading to lasting changes in brainwave patterns. This approach could complement or replace traditional treatments, especially when they fall short or have side effects. Providing clinicians with objective EEG metrics allows for continuous therapy evaluation and adjustment. If successful, this pilot could pave the way for broader clinical trials and, in the long run, be integrated into Danish psychiatric facilities to help those with auditory hallucinations. Overall, the study will be an important step towards developing more effective interventions for patients suffering from schizophrenia and serve to improve their clinical and functional prognosis.

Timeline

The project will adhere to the timeline displayed in the GANTT chart.

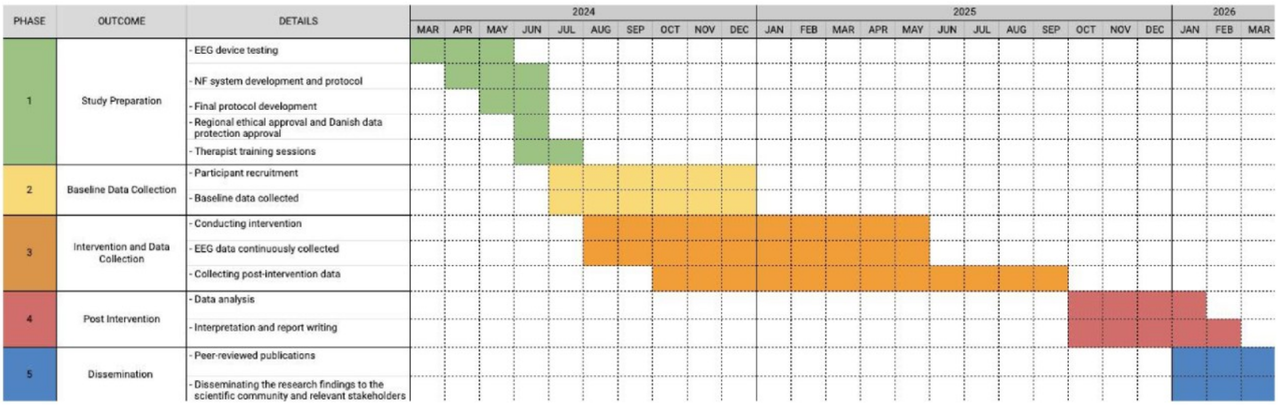

Dansk tillæg til forsøgsprotokol

Informeret samtykke og rekruttering

Rekruttering af patienter vil ske i samarbejde med sundhedspersonale fra de psykiatriske enheder i Region Hovedstadens Psykiatri eller i Region Sjælland. Personalet ambulante behandlingsenheder formidler kontakten til de projektansvarlige, såfremt patienten indvilliger heri.

For at rekruttere deltagere til projektet vil studiet også blive annonceret på Region Hovedstadens Psykiatris egen side, Forskningsportal Til Patientinklusion. Denne platform er specifikt designet til at præsentere igangværende forskningsprojekter for patienter og deres pårørende, som kunne være interesserede i at deltage i sådanne studier.
Følgende annoncetekst vil blive vist på portalen:

Neuro-VR

Et pilotstudie til undersøgelse af en innovativ virtual-reality baseret intervention med neurofeedback til at øge effekt og tolerabilitet ved psykose

Beskrivelse: Behandling af hørehallucinationer med brug af virtual reality (VR) har vist potentiale i tidligere forskningsprojekt. Her får deltagerne mulighed for at komme i dialog med en avatar af deres generende stemme, i håb om at de opnår mere kontrol i forholdet til stemmen. Neuro-VR undersøger nu, om denne terapiform, med samtidige målinger af hjerneaktivitet - såkaldt neurofeedback - kan være et redskab til at tilpasse terapien bedre til deltagernes individuelle behov samt at styrke arbejdet med følelsesregulering i mødet med stemmen.

Neuro-VR er et pilotstudie og et lodtrækningsforsøg, hvor alle, der inkluderes, får behandling. Den ene gruppe får 8 sessioner af VR-assisteret terapi, imens den anden gruppe får 8 sessioner af VR-assisteret terapi suppleret med neurofeedback. Neurofeedbacken foregår ved, at deltagerne får en EEG-hætte på i terapisessionerne, som vil måle på deres følelsesmæssige ubehag i mødet med stemmen.

Inklusionskriterier:

Alder 18-65 år

Skizofrenispektrum diagnose (ICD-10 kode: F20-F29)

Hørelseshallucinationer gennem de sidste tre måneder

Ingen ændret antipsykotisk medicinsk behandling 4 uger inden inklusion i projektet

Eksklusionskriterier:

Organisk hjernelidelse

IQ under 70

Primærdiagnose med afhængighed af stoffer/alkohol, der forhindrer deltagelse i terapien

Kan ikke udpege én dominant stemme at arbejde med

Hørehallucinationer på et andet sprog end dansk eller engelsk

Link til mere information her

Kontakt: Sara Soleim, mail: sara.breivik.soleim@regionh.dk

Studie ansvarlig: Louise Glenthøj, Seniorforsker, dr. med, ph.d, lektor, specialpsykolog og specialist i psykoterapi

Uanset rekrutteringsform er det en forudsætning, at patienten opfylder kriterierne for deltagelse i projektet, og at patientens primære behandler bliver informeret før projektstart.

Der fremsendes/overleveres skriftlig information om projektet minimum 48 timer før første fremmøde i VIRTU Research Group, Psykiatrisk Center København, og der informeres om retten til at medbringe en bisidder.

Under forløbet vil de forsøgsansvarlige have kontakt med patientens kontaktperson i f.eks. OPUS eller distriktspsykiatrisk center, såfremt patienten samtykker til dette. Det er en forudsætning, at patienten opfylder kriterierne for deltagelse i projektet før projektstart.

Ved fremmødet informeres patienten mundtlig om projektet af projektets forskningsassistent og denne svarer på eventuelle spørgsmål før patienten underskriver samtykke erklæringerne. Patienten tilbydes en betænkningstid på 24 timer efter at have modtaget mundtlig og skriftlig information om projektet. Patienter, der selv udtrykker ønske herom, kan samtykke med det samme. Pjecen ”Forsøgspersoners rettigheder i sundhedsvidenskabelige forskningsprojekter” udleveres sammen med deltagerinformationen.

Samtalen vil foregå i et aflukket lokale eventuelt sammen med patientens bisidder. I tilfælde af spørgsmål eller komplikationer vil patienten i hele forsøgsperioden have mulighed for telefonisk at kontakte de forsøgsansvarlige eller andre sundhedsfaglige personer tilknyttet VIRTU Research Group, Psykiatrisk Center København.

Under forløbet vil de forsøgsansvarlige have kontakt med patientens kontaktperson i psykiatrisk behandlingstilbud, såfremt patienten samtykker til dette.

Kontakthyppighed

Ved baseline samt 3 måneder efter behandlingsafslutning foretages assessment til vurdering af psykopatologi, funktionsniveau samt bivirkninger/negative hændelser. Hvis en forsøgsperson afbryder

protokollen, vil der blive tilbudt behandling efter relevante guidelines i psykiatrien i Region Hovedstaden eller Region Sjælland.

Kriterier for diskontinuation

Forsøgsdeltagerne kan når som helt forlade studiet, det er frivilligt at deltage. Hermed specificeret årsager til afbrydelse af studiet:

i. Frivillig afbrydelse af patienten selv; Patienten kan når som helst afbryde deltagelsen i forsøget uden at dette vil påvirke dennes videre behandling i sundhedssystemet.
ii. Forkert inklusion af patienten (f.eks. at patienten ikke opfylder inklusions/eksklusionskritererne).

Procedure for discontinuation

Patienter, som afbryder interventionen under indlæggelse, er fuldt berettiget til dette (jf. frivillighedsprincippet). Patienter, der ønsker at trække sig fra interventionen, vil blive undersøgt forelagt muligheden for at svare på spørgeskema omkring deres tilfredshed med behandlingen, hvis vedkommende ønsker dette.

Undersøgelser i studiet

Symptom- og funktionsniveau

Som anført i den engelske forsøgsprotokol, udføres assessments til vurdering af patienternes symptomniveau og funktionsniveau ved hjælp af interviews og selvrapporteringsskemaer. Yderligere stilles der mere kvalitative spørgsmål til deltagerne. De kvalitative spørgsmål omhandler deltagernes oplevelse af det at modtage terapi ved brug af virtual reality og brug af biofeedback samt hvad de oplevede var særligt effektivt eller kunne forbedres.

EEG-undersøgelse

Derudover foretages en en elektroencephalografi (EEG) undersøgelse ved start i projektet og ved behandlingsafslutning (3 måneders opfølgning), hvilket har til formål at undersøge, om der kan klarlægges eventuelle ændringer i hjerneaktivitet som resultat af behandlingen i projektet. EEG-undersøgelser er sikre og smertefrie og der er på nuværende tidspunkt ingen kendte bivirkninger eller helbredsrisici. Det er muligt at nogle forsøgspersoner vil føle sig let utilpasse når hætten med elektroderne påføres deres hoved, fordi

denne sidder lidt stramt til. Gelen som vi bruger til at forstærke EEG-optagelserne, er uden farve, lugtfri og allergivenlig. Undersøgelsen tager ca. 1½ time og gennemføres på Psykiatrisk Center Glostrup.

Tidsforbrug

Samlet tager undersøgelserne ca. 4 timer at gennemføre ved baseline og ca. 3 timer ved 3 måneders opfølgning.

Behandlingsmodaliteter

Den manualiserede, virtual reality intervention samt biofeedback er beskrevet i den engelske forsøgsprotokol.

Medicinering

Der tilbydes ikke medikamentel behandling som del af dette studium. Patienternes eventuelle psykofarmakologiske behandling varetages af patientens primærbehandler i deres psykiatriske tilbud. Såfremt en projektdeltager er i medikamentel behandling, og ved kontakt med projektet oplyser om bivirkninger hertil, vil man i forskningsprojektet rette henvendelse til patientens primærbehandler.

Forsøgets bivirkninger, risici og ulemper

Der kan være bivirkninger ved forsøget i form af ubehag ved at bruge virtual reality briller og hovedtelefoner og eventuelt oplevelse af at blive rundtosset eller køresyg, men generelt rapporteres der ikke bivirkninger til virtuel reality behandling[40]. Den afprøvede form for terapi, der er sammenlignelig med såkaldt avatarterapi, har udelukkende vist gavnlig effekt i forhold til at reducere patienternes symptomniveau og deres forpinthed og ubehag forbundet hermed[9,12], ligesom vi har positive erfaringer med denne behandling[15]. Der forventes derfor ikke at opstå bivirkninger eller negative hændelser som følge af interventionerne i dette studium. Der ses således ingen ulemper ved interventionerne. Skulle der imidlertid opstå bivirkninger eller negative hændelser som følge af terapien, vil det blive registreret og indberettet til Den Videnskabsetiske Komite. Se den engelske forsøgsprotokol for definition af bivirkninger/negative hændelser.

Databehandling

Projektet anmeldes til Datatilsynet. De forsøgsansvarlige vil indtaste patientens data direkte i en elektronisk CRF (Case Report Form) ved brug af dataindtastningssystemet REDCap. REDCap er et elektronisk dataindtastningssystem, der hostes af CIMT i Region Hovedstaden. REDCap er i overensstemmelse med den danske lovgivning for opbevaring af persondata (Datatilsynet). Data for hver patient er forbundet med et unikt løbenummer. Forsøgsansvarlige og forskningsassistenter er de eneste, der kan tilgå data i REDCap.

Projektet vil blive udført ved overholdelse af databeskyttelsesforordningen og databeskyttelsesloven.

Journaloplysninger

Indhentning af journaloplysninger forud for inklusion af forsøgsdeltager
I forbindelse med at sundhedspersonale i Region Hovedstadens Psykiatri eller Region Sjælland, formidler kontakten mellem potentielle forsøgspersoner og projektansvarlige, kan det være nødvendigt at få videregivet oplysninger fra patientens journal med henblik på at identificere om patienten kan indgå i projektet (dvs. opfylder inklusionskriterierne og ikke opfylder eksklusionskriterier). Der vil her være tale om, at sundhedspersonale videregiver journaloplysninger om potentielle forsøgsdeltager, før patienterne spørges om interesse for deltagelse, og derved før, at patienten har
afgivet samtykke til at deltage i forsøget. De specifikke helbredsforhold, der indhentes oplysninger om fra journalen, er symptomniveau (f.eks. omfang af psykosesymptomer), diagnoser, generelle helbredsforhold, funktionsniveau, medicinsk behandling, begavelsesniveau samt selvmordsrisiko.
Oplysningerne vil blive brugt til at afklare, hvorvidt patienten opfylder kriterierne for deltagelse, og efter denne vurdering, vil forsøgsansvarlige melde tilbage til sundhedspersonale om, hvorvidt patienten kan deltage i forsøget. Efter denne afklaring, vil sundhedspersonalet spørge patienten om interesse for deltagelse, og såfremt patienten indvilliger heri, vil kontakten mellem sundhedspersonale og forsøgsansvarlige blive formidlet.

Indhentning af journaloplysninger efter afgivet samtykke fra forsøgsdeltager
I forbindelse med, at forsøgspersoner spørges om interesse for deltagelse, vil de blive spurgt om at afgive samtykke til, at der kan indhentes journaloplysninger af relevans for projektet. Samtykket vil omfatte indhentning af samme oplysninger som i ovenstående: symptomniveau (f.eks. omfang af psykosesymptomer), diagnoser, generelle helbredsforhold, funktionsniveau, medicinsk behandling,

begavelsesniveau samt selvmordsrisiko), da disse kan være relevante at få adgang til, efter patienten er blevet spurgt om interesse for deltagelse, for at vurdere, hvorvidt patienten kan inkluderes i forsøget. Derudover vil samtykket omfatte indhentning af oplysninger om tidligere indlæggelser for psykiatriske eller somatiske tilstande, aktuel psykiatrisk behandling, aktuel og tidligere psykofarmakologisk behandling. Formålet med at indhente disse oplysninger er at afklare, hvorvidt der optræder en effekt af behandlingen eller det modsatte. Såfremt forsøgsdeltager har et igangværende behandlingsforløb i Region Hovedstadens Psykiatri i projektperioden, vil samtykket ligeledes omfatte indhentning af specifikke oplysninger fra patientjournal omkring varighed og intensitet af psykosesymptomer, medicinoplysninger, antal sessioner i det ambulante psykiatriske behandlingstilbud og lign.
Patienterne forventes at være grundigt informeret om projektet, når der formidles kontakt mellem patient og forsøgsansvarlige, hvorfor det vurderes, at størstedelen af patienterne vil være interesseret i at blive inkluderet. For dog at give et konservativt estimat vurderes det, at ud af 50 adspurgte vil 20 takke nej til projektdeltagelse på dette tidspunkt. Derfor estimeres det, at det vil dreje sig om 50 journaler. Tidsperioden for journaloplysninger er fra patientens initiale kontakt til det psykiatriske system, da der skal indhentes information om evt. organisk hjernelidelses-diagnose og diagnose med lavt begavelsesniveau.

Forsøgspersonens samtykke giver den forsøgsansvarlige, sponsor/sponsor repræsentant samt evt. kontrolmyndighed direkte adgang til at indhente relevante oplysninger i patientens journal, herunder elektronisk journal, med henblik på at se oplysninger om forsøgspersonens helbredsforhold, som er nødvendige som led i gennemførelse af forskningsprojekt samt i kontroløjemed, herunder egenkontrol, kvalitetskontrol og monitorering. Alle oplysninger som er indsamlet i projektet og fra journalen vil blive anvendt i projektet i anonymiseret form.

Håndtering af selvmordstanker/planer

Projektet følger god klinisk praksis for monitorering af selvmordsrisiko; herunder nyopståede eller forværring i selvmordstanker. Dette vurderes ved alle kliniske møder i projektet. Såfremt en patient oplever klinisk forværring og. eks. giver udtryk for nyopståede eller forværring i selvmordstanker, så er handleplanen at projektdeltagere, jf. det samtykke som er givet ved inklusion, straks tager kontakt til patientens behandlingsansvarlige kontaktperson i psykiatrien med henblik at vurdere behovet for at iværksætte relevante interventionstiltag (f.eks. intensivering af den ambulante behandling eller indlæggelse). Såfremt patienten ikke har et aktuelt forløb i psykiatrien, vil der blive taget kontakt til egen læge. Derudover vil det blive vurderet, om selvmordstankerne/evt. planer har en sådan karakter, at vedkommende skal følges til en vurdering og evt. indlæggelse i den psykiatriske modtagelse. Det vil også

blive vurderet, om man fra projektets side skal iværksætte henvisning til ambulant psykiatrisk behandling. Derudover vil der blive taget stilling til, om patienten på baggrund af forværringen må ekskluderes fra forsøget. Deltagelse i projektet er altid frivilligt, og samtykke kan trækkes tilbage uden at dette har nogen form for indflydelse for patientens rettigheder eller behandling.

Erstatningsordning

Forsøget er omfattet af patienterstatningen.

Videnskabsetisk redegørelse

Projektet vil blive anmeldt til Videnskabsetisk Komité og Videnscenter for Datasikkerhed.

Patienter som indgår i projektet, vil blive grundigt informeret både mundtligt og skriftligt, og deltagelse i forsøget accepteres først efter informeret skriftligt samtykke er afgivet. Det vil blive understreget overfor patienterne, at de til enhver tid og uden begrundelse kan trække sig fra projektet, og at dette ikke vil påvirke deres fremtidige behandling i sundhedssystemet.

Fordelene for patienterne

Patienterne vil gennemgå et udredningsprogram samt have tæt personlig kontakt til de forsøgsansvarlige. Yderligere vil alle patienter modtage en målrettet intervention, der sigter mod at dæmpe niveauet af hørehallucinationer ved psykose.

Behandlingen og eventuelle negative hændelser vil blive fulgt tæt. Behandlingen er planlagt således, at den følger gældende kliniske retningslinjer.

Patienterne vil få mulighed for at få tilbagemelding på de undersøgelser, de gennemgår, hvorfor det vil give patienterne, og deres behandlere, yderligere indsigt i patientens individuelle symptomer og vanskeligheder.

Ulemperne for patienter

Der vurderes primært at være en ulempe forbundet med forsøget i form af muligheden for at blive ”køresyg” ved brug af virtual reality headset. Dette er dog ikke en hyppig bivirkning, og den kan som oftest afsvækkes ved at tilbyde patienten en langsommere indføring i virtual reality scenarierne. Yderligere kan der være en ulempe forbundet med forsøget i form af tidsforbruget ved undersøgelserne (der foretages

ved baseline samt 3- måneders opfølgning). Tidsforbruget udgør dog kun ca. 3-4 timer undersøgelse pr. undersøgelse.

Studiets potentiale

Som angivet i engelsk forsøgsprotokol, er der ca. 25% af personer med psykose, der oplever vedvarende hørehallucinationer trods behandling med psykofarmaka. Derudover en betydelig gruppe, der oplever intolerable bivirkninger til psykofarmakologisk behandling, eller ikke ønsker denne behandling. Hørelseshallucinationerne er pinefulde og ubehagelige og begrænser personens evne til livsudfoldelse (f.eks. i forhold til daglige aktiviteter såsom at handle ind, begå sig social eller have job/uddannelse). Der er således et åbenlyst behov for at finde effektive, engagerende og tolerable terapiformer til denne patientgruppe. Dette studium afprøver effekten af en korterevarende, manualiseret, virtual reality-baseret terapiform i et klinisk forsøg. Hvis VR-behandlingen suppleret med biofeedback viser sig effektiv, kan den skabe udgangspunkt for at afprøve den i et stort randomiseret klinisk forsøg mhp at skabe evidens og på sigt mulig implementering i relevante behandlingsenheder under tæt optræning og supervision.

Økonomiske forhold og klausuler

Forsøget har modtaget støtte i form af bevilling på 2.000.000 kr. fra Lundbeckfonden.

Beløbet skal bruges til aflønning af to forskningsassistenter i projektet samt psykolog, der udfører VR-behandlingen samt integrering af neurofeedback data i VR-programmet. Virtual reality-programmet er udviklet af den Heka VR, en dansk privatejet virksomhed, der er specialiseret i VR-løsninger. Heka VR har ingen indflydelse på studiets design, dataindsamling, analyser eller præsentation af data, ligesom Heka VR heller ikke har adgang til projektets data. Heka VR har således ikke habilitetsproblemer i forhold til studiet. Forsøgsansvarlig har ingen økonomisk interesse til Heka VR.

Initiativtager

Forskningsleder, specialpsykolog, dr.med, ph.d., lektor Louise Birkedal Glenthøj fra VIRTU Research Group på Psykiatrisk Center København har taget initiativ til at iværksætte projektet. VIRTU er i forvejen særdeles aktiv i forskning i virtual reality behandling i psykiatrien.

Vederlag

Patienter vil ikke modtage nogen godtgørelse, idet de er under behandling. De vil få godtgjort deres transportudgifter og få forplejning på undersøgelsesdagene.

Offentliggørelse

Såvel positive som negative og inkonklusive forskningsresultater vil blive offentliggjort i internationale tidsskrifter. Resultaterne vil ligeledes blive præsenteret på nationale og internationale møder og kongresser.

Projektet vil blive registreret på www.clinicaltrials.gov,når der foreligger godkendelse fra Videnskabsetisk Komité samt Videnscenter for Dataanmeldelser, og inden første patient er inkluderet i projektet.

Organisation

Psykiatrisk Center København stiller kontorlokaler til rådighed. Her vil interviews omkring symptomer-funktionsniveau foregå og blive udført af forsøgsansvarlige psykologer og læger. Hovedansvaret for behandlingen vil blive varetage af psykolog med specialviden inden for feltet. Projektgruppen består af forskningsleder og lektor PhD, dr.med. Louise Birkedal Glenthøj, professor Bjørn Ebdrup, Seniorforsker Tina Dam Kristensen, post.doc Melissa Larsen.

Gennemførlighed

Forskergruppen har stor erfaring med interventionsforsøg til patienter med psykiatriske lidelser og har nyligt afsluttet verdens største randomiserede kliniske forsøg til undersøgelse af VR-behandling til hørehallucinationer. Der er er et godt og tæt samarbejde med kliniske forskningsenheder i Region Hovedstadens Psykiatri, der gerne henviser patienter til projektet. Baseret på, at vi netop har inkluderet 266 personer med hørehallucinationer i lignende forskningsprojekt, synes det realistisk at inkludere 30 personer i dette projekt.

References:

1. World Health Organization. Schizophrenia [Internet]. [cited 2023 Aug 10]. Available from: https://www.who.int/news-room/fact-sheets/detail/schizophrenia

2. Rössler W, Joachim Salize H, Van Os J, Riecher-Rössler A. Size of burden of schizophrenia and psychotic disorders. Eur Neuropsychopharmacol. 2005 Aug;15(4):399–409.

3. Waters F, Allen P, Aleman A, Fernyhough C, Woodward TS, Badcock JC, et al. Auditory Hallucinations in Schizophrenia and Nonschizophrenia Populations: A Review and Integrated Model of Cognitive Mechanisms. Schizophr Bull. 2012 Jul 1;38(4):683–93.

4. Mayor S. Avatar therapy reduces auditory hallucinations in schizophrenia, trial finds. BMJ. 2017 Nov 23;j5458.

5. Wils RS, Gotfredsen DR, Hjorthøj C, Austin SF, Albert N, Secher RG, et al. Antipsychotic medication and remission of psychotic symptoms 10 years after a first-episode psychosis. Schizophr Res. 2017 Apr;182:42–8.

6. Gustavsson A, Svensson M, Jacobi F, Allgulander C, Alonso J, Beghi E, et al. Cost of disorders of the brain in Europe 2010. Eur Neuropsychopharmacol. 2011 Oct;21(10):718–79.

7. Candida M, Campos C, Monteiro B, Rocha NBF, Paes F, Nardi AE, et al. Cognitive-behavioral therapy for schizophrenia: an overview on efficacy, recent trends and neurobiological findings. Med Express [Internet]. 2016 [cited 2025 Mar 28];3(5). Available from: http://www.gnresearch.org/doi/10.5935/MedicalExpress.2016.05.01

8. Van Der Gaag M, Valmaggia LR, Smit F. The effects of individually tailored formulation-based cognitive behavioural therapy in auditory hallucinations and delusions: A meta-analysis. Schizophr Res. 2014 Jun;156(1):30–7.

9. Craig TK, Rus-Calafell M, Ward T, Leff JP, Huckvale M, Howarth E, et al. AVATAR therapy for auditory verbal hallucinations in people with psychosis: a single-blind, randomised controlled trial. Lancet Psychiatry. 2018 Jan;5(1):31–40.

10. Paulik G. The Role of Social Schema in the Experience of Auditory Hallucinations: A Systematic Review and a Proposal for the Inclusion of Social Schema in a Cognitive Behavioural Model of Voice Hearing. Clin Psychol Psychother. 2012 Nov;19(6):459–72.

11. Leff J, Williams G, Huckvale MA, Arbuthnot M, Leff AP. Computer-assisted therapy for medication-resistant auditory hallucinations: proof-of-concept study. Br J Psychiatry. 2013 Jun;202(6):428–33.

12. Percie Du Sert O, Potvin S, Lipp O, Dellazizzo L, Laurelli M, Breton R, et al. Virtual reality therapy for refractory auditory verbal hallucinations in schizophrenia: A pilot clinical trial. Schizophr Res. 2018 Jul;197:176–81.

13. Dellazizzo L, Potvin S, Phraxayavong K, Dumais A. One-year randomized trial comparing virtual reality-assisted therapy to cognitive–behavioral therapy for patients with treatment-resistant schizophrenia. Npj Schizophr. 2021 Feb 12;7(1):9.

14. Vernal DL, Nordentoft M, Christensen MJ, Smith LC, Mariegaard L, Mainz J, et al. Status and Clinical Experiences from the Challenge Trial – A Randomized Controlled Trial Investigating Virtual Reality-based Therapy for Auditory Hallucinations. World Soc Psychiatry. 2023 Jan;5(1):71–6.

15. Huang W, Wu W, Lucas MV, Huang H, Wen Z, Li Y. Neurofeedback Training With an Electroencephalogram-Based Brain-Computer Interface Enhances Emotion Regulation. IEEE Trans Affect Comput. 2023 Apr 1;14(2):998–1011.

16. Kothgassner OD, Goreis A, Bauda I, Ziegenaus A, Glenk LM, Felnhofer A. Virtual reality biofeedback interventions for treating anxiety: A systematic review, meta-analysis and future perspective. Wien Klin Wochenschr. 2022 Jan;134(S1):49–59.

17. Schwartz GE. Biofeedback as therapy: Some theoretical and practical issues. Am Psychol. 1973;28(8):666–73.

18. Ahrweiler N, Santana-Gonzalez C, Zhang N, Quandt G, Ashtiani N, Liu G, et al. Neural Activity Associated with Symptoms Change in Depressed Adolescents following Self-Processing Neurofeedback. Brain Sci. 2022 Aug 25;12(9):1128.

19. Van Doren J, Arns M, Heinrich H, Vollebregt MA, Strehl U, K. Loo S. Sustained effects of neurofeedback in ADHD: a systematic review and meta-analysis. Eur Child Adolesc Psychiatry. 2019 Mar;28(3):293–305.

20. Amico F, Keane M, Lee M, McCarthy-Jones S. A Feasibility Study of LORETA Z-Score Neurofeedback Training in Adults with Schizophrenia-Spectrum Disorder Experiencing Treatment-Resistant Auditory Verbal Hallucinations. NeuroRegulation. 2022 Sep 30;9(3):135–46.

21. Vernon DJ. Can Neurofeedback Training Enhance Performance? An Evaluation of the Evidence with Implications for Future Research. Appl Psychophysiol Biofeedback. 2005 Dec;30(4):347–64.

22. McCarthy-Jones S. Taking Back the Brain: Could Neurofeedback Training Be Effective for Relieving Distressing Auditory Verbal Hallucinations in Patients With Schizophrenia? Schizophr Bull. 2012 Jul 1;38(4):678–82.

23. Rosenfeld JP, Baehr E, Baehr R, Gotlib IH, Ranganath C. Preliminary evidence that daily changes in frontal alpha asymmetry correlate with changes in affect in therapy sessions. Int J Psychophysiol. 1996 Aug;23(1–2):137–41.

24. Sherlin LH, Arns M, Lubar J, Heinrich H, Kerson C, Strehl U, et al. Neurofeedback and Basic Learning Theory: Implications for Research and Practice. J Neurother. 2011 Oct;15(4):292–304.

25. Teplan M. Fundamentals of EEG measurement. Meas Sci Rev. 2002(2):1–11.

26. Al-Kadi M, Reaz M, Ali M. Evolution of Electroencephalogram Signal Analysis Techniques during Anesthesia. Sensors. 2013 May 17;13(5):6605–35.

27. Tandle A, Jog N, D´cunha P, Chheta M. Classification of artefacts in EEG signal recordings and overview of removing techniques. Int J Comput Appl. 2015(975):8887.

28. Ismail LE, Karwowski W. Applications of EEG indices for the quantification of human cognitive performance: A systematic review and bibliometric analysis. Mumtaz W, editor. PLOS ONE. 2020 Dec 4;15(12):e0242857.

29. Sritharan A, Line P, Sergejew A, Silberstein R, Egan G, Copolov D. EEG coherence measures during auditory hallucinations in schizophrenia. Psychiatry Res. 2005 Sep;136(2–3):189–200.

30. Ishii R, Shinosaki K, Ikejiri Y, Ukai S, Yamashita K, Iwase M. Theta rhythm increases in left superior temporal cortex during auditory hallucinations in schizophrenia: a case report. NeuroReport. 11/2000(14):3283–7.

31. Juszczak G. The low-frequency oscillation model of hallucinations in neurodegenerative disorders and in delirium. Iran J Med Hypotheses Ideas. 5/2011:11.

32. Nayani TH, David AS. The auditory hallucination: a phenomenological survey. Psychol Med. 1996 Jan;26(1):177–89.

33. Surmeli T, Ertem A, Eralp E, Kos IH. Schizophrenia and the Efficacy of qEEG-Guided Neurofeedback Treatment: A Clinical Case Series. Clin EEG Neurosci. 2012 Apr;43(2):133–44.

34. Liu Y, Du S. Psychological stress level detection based on electrodermal activity. Behav Brain Res. 2018 Apr;341:50–3.

35. Di Lascio E, Gashi S, Santini S. Laughter Recognition Using Non-invasive Wearable Devices. In: Proceedings of the 13th EAI International Conference on Pervasive Computing Technologies for Healthcare [Internet]. Trento Italy: ACM; 2019 [cited 2025 Mar 18]. p. 262–71. Available from: https://dl.acm.org/doi/10.1145/3329189.3329216

36. Di Lascio E, Gashi S, Santini S. Unobtrusive Assessment of Students' Emotional Engagement during Lectures Using Electrodermal Activity Sensors. Proc ACM Interact Mob Wearable Ubiquitous Technol. 2018 Sep 18;2(3):1–21.

37. Bradley MM, Lang PJ. Emotion and Motivation. In: Cacioppo JT, Tassinary LG, Berntson G, editors. Handbook of Psychophysiology [Internet]. 3rd ed. Cambridge: Cambridge University Press; 2007 [cited 2025 Mar 18]. p. 581–607. Available from: http://ebooks.cambridge.org/ref/id/CBO9780511546396A034

38. Kalimeri K, Saitis C. Exploring multimodal biosignal features for stress detection during indoor mobility. In: Proceedings of the 18th ACM International Conference on Multimodal Interaction [Internet]. Tokyo Japan: ACM; 2016 [cited 2025 Mar 18]. p. 53–60. Available from: https://dl.acm.org/doi/10.1145/2993148.2993159

39. Hernandez J, Morris RR, Picard RW. Call Center Stress Recognition with Person-Specific Models. In: D'Mello S, Graesser A, Schuller B, Martin JC, editors. Affective Computing and Intelligent Interaction [Internet]. Berlin, Heidelberg: Springer Berlin Heidelberg; 2011 [cited 2025 Mar 18]. p. 125–34. (Lecture Notes in Computer Science; vol. 6974). Available from: http://link.springer.com/10.1007/978-3-642-24600-5_16

40. Reinhardt T, Schmahl C, Wüst S, Bohus M. Salivary cortisol, heart rate, electrodermal activity and subjective stress responses to the Mannheim Multicomponent Stress Test (MMST). Psychiatry Res. 2012 Jun;198(1):106–11.

41. Benedek M, Kaernbach C. A continuous measure of phasic electrodermal activity. J Neurosci Methods. 2010 Jun;190(1):80–91.

42. Meuret AE, Rosenfield D, Wilhelm FH, Zhou E, Conrad A, Ritz T, et al. Do Unexpected Panic Attacks Occur Spontaneously? Biol Psychiatry. 2011 Nov;70(10):985–91.

43. Mevlevioğlu D, Tabirca S, Murphy D. Real-Time Classification of Anxiety in Virtual Reality Therapy Using Biosensors and a Convolutional Neural Network. Biosensors. 2024 Mar 3;14(3):131.

44. Charlton PH, Celka P, Farukh B, Chowienczyk P, Alastruey J. Assessing mental stress from the photoplethysmogram: a numerical study. Physiol Meas. 2018 May 15;39(5):054001.

45. Smith S, Maisrikrod S. Wearable Electrocardiogram Technology: Help or Hindrance to the Modern Doctor? JMIR Cardio. 2025 Feb 10;9:e62719.

46. Lüddecke R, Felnhofer A. Virtual Reality Biofeedback in Health: A Scoping Review. Appl Psychophysiol Biofeedback. 2022 Mar;47(1):1–15.

47. Lee H, Choi J, Jung D, Hur JW, Cho CH. The Effects of Virtual Reality Treatment on Prefrontal Cortex Activity in Patients With Social Anxiety Disorder: Participatory and Interactive Virtual Reality Treatment Study. J Med Internet Res. 2021 Dec 17;23(12):e31844.

48. Pallavicini F, Algeri D, Repetto C, Gorini A, Riva G. Biofeedback, virtual reality and mobile phones in the treatment of generalized anxiety disorder (GAD): A phase-2 controlled clinical trial. J Cyber Ther Rehabil. 2009;2(4):315–27.

49. Bossenbroek R, Wols A, Weerdmeester J, Lichtwarck-Aschoff A, Granic I, Van Rooij MMJW. Efficacy of a Virtual Reality Biofeedback Game (DEEP) to Reduce Anxiety and Disruptive Classroom Behavior: Single-Case Study. JMIR Ment Health. 2020 Mar 24;7(3):e16066.

50. Gorini Alessandra, Pallavicini Federica, Algeri Davide, Repetto Claudia, Gaggioli Andrea, Riva Giuseppe. Virtual Reality in the Treatment of Generalized Anxiety Disorders. In: Studies in Health Technology and Informatics [Internet]. IOS Press; 2010 [cited 2025 Mar 31]. Available from: https://www.medra.org/servlet/aliasResolver?alias=iospressISSNISBN&issn=0926-9630&volume=154&spage=39

51. Oh SH, Park JW, Cho SJ. Effectiveness of the VR Cognitive Training for Symptom Relief in Patients with ADHD. J Web Eng [Internet]. 2022 Mar 22 [cited 2025 Mar 28]; Available from: https://journals.riverpublishers.com/index.php/JWE/article/view/11997

52. Fan J, Wade JW, Key AP, Warren ZE, Sarkar N. EEG-Based Affect and Workload Recognition in a Virtual Driving Environment for ASD Intervention. IEEE Trans Biomed Eng. 2018 Jan;65(1):43–51.

53. Bhakta S, Gonzalez C, Wu Y, Din JM, Minhas J, Talledo J, et al. 210. Feasibility and Reliability of a Combined Virtual Reality - Electroencephalogram Paradigm to Assess Sensory Information Processing in Schizophrenia Patients. Biol Psychiatry. 2023 May;93(9):S178–9.

54. Rus-Calafell M, Garety P, Sason E, Craig TJK, Valmaggia LR. Virtual reality in the assessment and treatment of psychosis: a systematic review of its utility, acceptability and effectiveness. Psychol Med. 2018 Feb;48(3):362–91.

55. Henshall KR, Sergejew AA, Rance G, McKay CM, Copolov DL. Interhemispheric EEG coherence is reduced in auditory cortical regions in schizophrenia patients with auditory hallucinations. Int J Psychophysiol. 2013 Jul;89(1):63–71.

56. Bell ML, Whitehead AL, Julious SA. Guidance for using pilot studies to inform the design of intervention trials with continuous outcomes. Clin Epidemiol. 2018 Jan;Volume 10:153–7.
