# Supplementary material for: Study protocol for a randomized clinical pilot trial investigating feasibility and efficacy of augmenting a virtual reality-assisted intervention targeting auditory verbal hallucinations with biofeedback: The Neuro-VR study
Source: PLoS One. 2026 Feb 26;21(2):e0333716. doi: 10.1371/journal.pone.0333716 (PMC12944719; doi:10.1371/journal.pone.0333716)
Supplement: S1 Fig — Schedule showing the timing of participant enrolment, interventions, and assessments. (PDF) [file pone.0333716.s003.pdf]

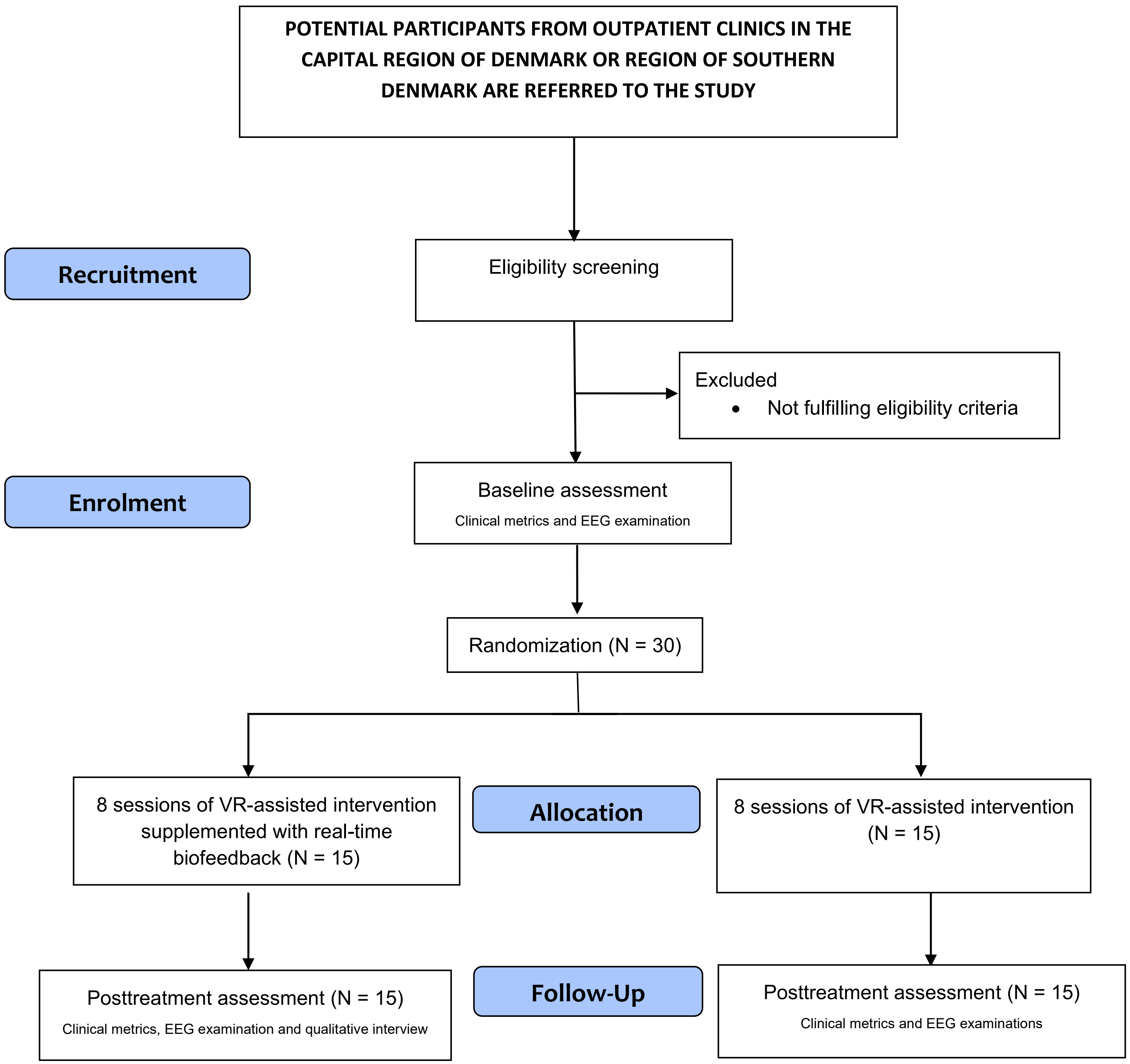


**S1 Fig. CONSORT 2025 Flow Diagram.** Schedule showing the timing of participant enrolment, interventions, and assessments.
